# Implicit and explicit avoidance in sexual trauma victims suffering from posttraumatic stress disorder: a pilot study

**DOI:** 10.3402/ejpt.v5.21359

**Published:** 2014-02-11

**Authors:** Pascal Fleurkens, Mike Rinck, Agnes van Minnen

**Affiliations:** 1 Behavioural Science Institute, Radboud University Nijmegen, Nijmegen, The Netherlands; 2 Overwaal, Centre for Anxiety Disorders, Nijmegen, The Netherlands

**Keywords:** PTSD, avoidance, Approach-Avoidance Task (AAT), trauma-relatedness

## Abstract

**Background:**

Avoidance of stimuli that are associated with the traumatic event is a key feature of posttraumatic stress disorder (PTSD). Thus far, studies on the role of avoidance in the development and maintenance of PTSD focused primarily on strategic or explicit avoidance. However, patients may also show implicit avoidance behavior, which may remain even when explicit avoidance is reduced.

**Objectives:**

The present pilot study was designed to test the hypothesis that PTSD patients show implicit avoidance of threatening, trauma-related stimuli. In addition, it was tested whether this avoidance behavior also occurs for other stimuli.

**Methods:**

The Approach-Avoidance Task was used as an indirect measure of avoidance. Participants were 16 women suffering from PTSD who had experienced a sexual trauma, and 23 healthy non-traumatized women. Using a joystick, they pulled pictures closer to themselves or pushed them away. The pictures varied in content, being either high-threat sexual, non-threat sexual, high-threat accident, or positive.

**Results:**

Compared to control participants, PTSD patients avoided high-threat sexual pictures, and the degree of avoidance was predicted by self-reported arousal level. Moreover, PTSD patients with high levels of self-reported explicit avoidance, depressive symptoms, and PTSD symptom severity also avoided high-threat accident pictures.

**Conclusions:**

These findings point to the possible importance of threat value instead of trauma-relatedness in explaining implicit avoidance. The results are discussed in light of cognitive-behavioral models of PTSD, and clinical implications are suggested.

After experiencing a threatening event, such as a car accident, rape, or a violent act, some people develop posttraumatic stress disorder (PTSD; DSM IV-R, APA, 2000) at some point in their lives (see De Vries & Olff, 2009). Although prevalence rates vary largely, ranging from 1.9% in Europe (almost no PTSD in Switzerland and 7.4% in the Netherlands) to 6.8% in the United States and 37.4% in postconflict countries (for an overview, see De Vries & Olff, 2009), it can be stated that PTSD is a common psychiatric disorder. Avoidance of stimuli associated with the traumatic event is a key feature of this disorder (Ehlers & Clark, 2000; Foa & Kozak, 1986) and is, therefore, addressed in the present study. Avoidance of threatening stimuli serves an important function for PTSD patients: It prevents recollections of the traumatic event, and thereby the negative and fearful thoughts, feelings and cognitions associated with the event. According to Lohr, Olantuji, and Sawchuk (2007), recollections of the trauma serve as a signal of danger, which provokes high levels of anxiety. By avoiding recollections of the trauma, patients protect themselves from danger and being harmed again. Due to this avoidance, however, patients also prevent themselves from learning new response patterns because they do not fully subject themselves to the emotional processing of their anxiety (Foa, Huppert, & Cahill, 2006; Foa & Kozak, 1986). In line with this, the cognitive model of Ehlers and Clark (2000) states that avoidance is a maladaptive control strategy that prevents disconfirmation of negative appraisals, resulting in maintenance of perceived current threat.

In line with this, trauma-focused treatments stress the role of avoidance in the maintenance of PTSD. Prolonged exposure to safe but anxiety-provoking trauma-related stimuli is considered a treatment of choice for PTSD (Ballenger et al., 2004; Nemeroff et al., 2006), and it is recommended worldwide in official PTSD treatment guidelines, for instance, by the International Society for Traumatic Stress Studies (Foa, Keane, Friedman, & Cohen, 2009) or the National Institute for Health and Care Excellence, Clinical Guidelines on PTSD (NICE, 2005).

Thus far, however, studies on the role of avoidance in the development and maintenance of PTSD focused primarily on strategic or explicit avoidance, as reported by the patients in questionnaires or interviews (see, for instance, Van Minnen & Hagenaars, 2010). Although these measures can be very useful for investigating behavioral avoidance symptoms in PTSD, they also have significant disadvantages, namely that they reflect mainly controlled processes, instead of addressing implicit aspects of behavior. In the following, we will refer to this avoidance behavior as *explicit avoidance*. In addition, patients may not be aware of some of their avoidance behavior because they avoid situations and activities in an implicit way (see, e.g., Foa, Hembree, & Rothbaum, 2007; Taylor, 2006). However, *implicit avoidance* behavior is very difficult, if not impossible, to grasp and report because patients are frequently not aware of it.

This calls for indirect measures, so that implicit processes can be studied (Buckley, Blanchard, & Neill, 2000). One such indirect measure is the Approach-Avoidance Task (AAT; Rinck & Becker, 2007). In the AAT, participants face a screen on which a picture is displayed. Participants have to react to the picture by pushing it away from themselves (indicating avoidance) or pulling it toward themselves (indicating approach) by means of a joystick connected to the computer. Whether the pictures have to be pulled closer or pushed away is usually determined by a stimulus feature unrelated to picture contents, for example, format, tilt, or frame color. This indirect version of the AAT is aimed at measuring implicit action tendencies because the stimulus feature of interest (e.g., its emotional valence) is task irrelevant. If the feature of interest affects responses nevertheless (e.g., when pleasant pictures are approached more quickly, while unpleasant ones are avoided more quickly), even though participants are not asked to pay attention to it, it is taken as evidence for partly automatic processing of the feature.

With the AAT, implicit approach-avoidance tendencies toward pleasant and unpleasant pictures have been assessed in various disorders (e.g., fear of spiders: Klein, Becker, & Rinck, 2011; Rinck & Becker, 2007; social anxiety: Heuer, Rinck, & Becker, 2007; Lange, Keijsers, Becker, & Rinck, 2008; Roelofs et al., 2010; pathological skin picking: Schuck, Keijsers, & Rinck, 2012; addictions: Wiers, Eberl, Rinck, Becker, & Lindenmeyer, 2011; Wiers, Rinck, Dictus, & Van den Wildenberg, 2009). However, to our knowledge no studies on implicit avoidance behavior of PTSD patients have been reported yet, making the present study the first with this objective.

The major goal of the present study was to investigate implicit avoidance tendencies in PTSD patients who had suffered sexual trauma, using the AAT as an indirect measure of avoidance. More specifically, we studied which kind of distinct topics would lead to implicit approach or avoidance tendencies. Some stimuli, for example, were expected to elicit anxiety and subsequent implicit avoidance in PTSD patients, because they were highly threatening and trauma-related. Here, we call these *high-threat sexual pictures* (e.g., a sexual assault scene). Other stimuli were expected to be potentially anxiety-provoking for those who experienced sexual trauma, but not for other people. Here, we call these *non-threat sexual pictures* (e.g., a love scene). The latter stimuli are neutral or positive in nature, but their content is related to the experienced trauma, possibly causing higher anxiety levels and subsequent implicit avoidance.

The latter notion is in line with an explanation that has been proposed to explain attentional biases for positive words in PTSD patients, arguing that the words were related to the same topic as the experienced trauma, like “love” or “enchantment” in case of rape victims (Cassiday, McNally, & Zeitlin, 1992; Paunovic, Lundh, & Öst, 2002). In this sense, trauma-relatedness may be more important for PTSD-related cognitive biases than the threatening nature of the materials, in line with the results of a recent study by Fleurkens, Rinck, and Van Minnen (2011). This study showed that only trauma-related materials, rather than threatening materials in general, caused distraction and attentional bias in an Emotional Stroop Task (EST), especially in those PTSD patients who experienced more severe arousal symptoms. However, it would be premature to generalize from EST findings to other cognitive tasks, because they assess different processes. While the EST assesses attention biases, measured by *generally* increased reaction times in any response to disorder-related stimuli, the AAT assesses the relative strength of action tendencies, measured as increased reaction times of specific responses, here pull versus push movements. Thus, neither the underlying processes (attention vs. approach-avoidance) nor the analytical approach (analyzing mean RTs vs. pull-push RT differences) are comparable.

The implicit avoidance tendencies studied here might also occur for pictures that are *un*related to trauma, but are nonetheless threatening. This notion would be in line with findings of attentional interference effects for both trauma-related and unrelated high-threat words (Litz et al., 1996). It would also be in accordance with the findings of increased amygdala activation—indicating enhanced emotional processing—to masked trauma-unrelated fearful faces in PTSD patients (Rauch et al., 2000), and in motor vehicle accident survivors with acute PTSD (Armony, Corbo, Clément, & Brunet, 2005). The latter effect occurred as a function of PTSD symptom severity. To test whether threatening stimuli that are not related to the experienced trauma are also avoided by PTSD patients, so-called *high-threat accident pictures* were also included in the present study (e.g., a car accident scene). Finally, *positive pictures* (e.g., a scene with flowers) were included as a positive control condition to test whether emotional stimuli in general would cause implicit avoidance in PTSD patients.

In sum, the present study was designed to test the hypothesis that PTSD patients show implicit avoidance of threatening, trauma-related stimuli (e.g., sexual assault scenes). In addition, it was tested whether this avoidance behavior would also occur in response to stimuli that are also trauma-related, but non-threatening (e.g., love scenes), in response to stimuli that are also threatening, but trauma-unrelated (e.g., car accidents), and in response to stimuli that are neither threatening nor trauma-related (e.g., flowers). These questions were addressed by comparing the PTSD patients’ implicit approach-avoidance behavior to that of the control participants. Moreover, we assessed whether the strength and width of the PTSD patients’ avoidance behavior depended on variables that are known to be related to PTSD: level of explicit avoidance behavior, severity of different PTSD symptoms, and symptoms of depression and dissociation. These variables were assessed with the questionnaires described below.

## Methods

### Participants

Two groups were included in the present study.^1^ Group 1 consisted of 16 female patients (mean age 34.4 years, *SD*=10.5) who met DSM-IV-TR (APA, 2000) criteria for chronic PTSD, established by structured clinical interviews (Clinician Administered PTSD Scale [CAPS]; Blake et al., 1995; Dutch version: Hovens, Luinge, & Van Minnen, 2005). The patients were referrals to an outpatient clinic specializing in the treatment of anxiety disorders and an outpatient psychotherapy clinic. All patients had experienced sexual trauma and had not experienced or witnessed a traumatic traffic accident. In addition to PTSD, 11 patients met one or more secondary DSM-IV Axis 1 diagnoses: mood disorder (*n=*8), anxiety disorder (*n=*10), eating disorder (*n=*2), or other (*n=*1). Group 2 consisted of 23 healthy female control participants (mean age 40.2 years, *SD*=11.9) who had not experienced sexual trauma, nor had they experienced or witnessed a traumatic traffic accident. Controls were selected based on age and educational level, considering the composition of the PTSD group. This procedure was successful: Two one-way ANOVAs revealed no significant differences between the two groups in age, *F*(1,38)=2.49, *ns*, or educational level, *F*(1,38)=1.65, *ns*.

### General procedure

PTSD patients participated in a larger ongoing randomized controlled treatment study with a heterogeneous trauma population. Only those who had experienced sexual trauma and who had PTSD as primary diagnosis were selected for the present study. Exclusion criteria were non-native Dutch speaking, color-blindness, dyslexia, and current alcohol or drug abuse. PTSD patients were assessed for the present study before treatment. After inclusion and giving informed consent, they filled in several questionnaires (see Measures section below). Then they completed an EST^2^ and the AAT (order was randomized), while the experimenter stayed in the room to check whether patients performed the task correctly.

All controls were interviewed and screened for exclusion criteria by the first author. Controls were screened for symptoms of PTSD, major depressive disorder, psychotic disorder, panic disorder, and current alcohol or drug abuse. Furthermore, they were screened for a history of experiencing or witnessing sexual trauma or a traumatic car accident. It was checked whether Dutch was their native language, whether their color vision was unimpaired, and whether they were not dyslexic. After inclusion and giving informed consent, the controls filled in several measurements and completed an EST and the AAT (the same as the PTSD patients, see above) at their home or workplace. They received a cheque worth 7.50 € for participating. The total duration of the experiment was approximately 1 hour. Both PTSD patients and controls were informed that they could stop the experiment at any time. After the experiment, both groups were elaborately debriefed.

### Measures

#### PTSD symptom severity

As stated earlier, in the patient group, PTSD diagnosis was established with the Dutch translation of CAPS (Blake et al., 1995; Dutch version: Hovens, Luinge, & Van Minnen, 2005), a semistructured interview. With the CAPS, both frequency and intensity of posttraumatic symptoms can be measured. To assess self-reported PTSD symptom severity, patients completed the PTSD Symptom Scale Self-Report (PSS-SR) (Foa Riggs, Dancu, & Rothbaum, 1993; Dutch version: Arntz, 1993). The PSS-SR items measure the frequency of 17 DSM-IV-TR symptoms of PTSD. The scale consists of three subscales: re-experiencing (items 1 to 5), avoidance and numbing (items 6 to 12; items 6 and 7 measure avoidance, items 8 to 12 measure numbing), and arousal (items 13 to 17). Its test–retest reliability and internal consistency are described as good (Foa et al., 1993). The psychometric properties of the Dutch version have been described as good (Engelhard, Arntz, & Van den Hout, 2007; Wohlfarth, Van den Brink, Winkel, & Ter Smitten, 2003).

#### Psychopathology

Depressive symptoms were assessed with the Beck Depression Inventory (BDI) (Beck, Ward, Mendelsohn, Mock, & Erbaugh, 1961; Dutch version: Van der Does, 2002). The BDI is a 21-item inventory designed to measure depressed mood and other symptoms of depression. The inventory has excellent psychometric properties (Beck, Steer, & Garbin, 1988). As an index of dissociative symptoms, the Dissociative Experience Scales (DES) (Bernstein & Putnam, 1986) was used. The DES is a 28-item questionnaire measuring dissociative symptoms. Its reliability and validity have been described as good (Bernstein & Putnam, 1986).

#### Explicit avoidance

To identify explicit avoidance behavior, participants completed the Posttraumatic Avoidance Behaviour Questionnaire (PABQ) (Van Minnen & Hagenaars, 2010). The PABQ is a 25-item inventory, consisting of seven subscales measuring typical situations and activities that PTSD patients avoid. Its internal consistency, test–retest reliability, convergent, and discriminative validity are considered good (Van Minnen & Hagenaars, 2010).

With the exception of the CAPS, the controls completed the same questionnaires as the patients did: PSS-SR, PABQ, DES, and BDI.

### Implicit avoidance: AAT

The AAT has been used before to assess implicit avoidance tendencies in anxiety disorders (e.g., Rinck & Becker, 2007). In a study concerning implicit avoidance of spiders, the internal consistency of the AAT has been described as potentially high for a reaction time task, with Cronbach's alpha of .59 (Reinecke, Becker, & Rinck, 2010). For the present AAT, eight *high-threat sexual* pictures and eight *non-threat sexual* pictures were selected. Furthermore, we selected eight *high-threat accident* pictures and eight *non-threat vehicle* pictures. Finally, eight positive pictures were selected, yielding a total of 5×8=40 pictures.^3^ All pictures were selected from the International Affective Picture System (IAPS) (Bradley & Lang, 2007; Lang, Bradley, & Cuthbert, 1999) and from the Internet. The threatening nature of the pictures and their relatedness to trauma were assessed in a pilot study, using a group of non-traumatized students. We found that the high-threat sexual pictures were highly threatening and related to sexual trauma (e.g., an assault scene). The non-threat sexual pictures consisted of scenes that were neutral or positive in nature, but content-related to sexual trauma (e.g., a love scene). High-threat accident pictures were highly threatening, but related to traffic accidents rather than sexual trauma (e.g., a car accident). The positive pictures (e.g., flowers) were non-threatening and not related to either trauma type at all.

In the present AAT, all pictures were presented on a computer screen with a resolution of 1,024×768 pixels. Participants had to respond to the pictures by pulling a joystick connected to the computer toward themselves or pushing it away with their dominant hand. All pictures had a colored frame (white vs. red), and the correct response (pulling or pushing) depended on frame color. Picture content was irrelevant to the instructions. Half of the participants in each group had to pull the pictures with a white frame and push the pictures with a red frame, and vice versa for the other participants. Each of the 40 pictures was presented with both frame colors, such that each participant had to push it twice and pull it twice, yielding a total of 160 picture presentations in a randomized order. Pictures disappeared, irrespective of the correctness of the movement, when the joystick was pushed or pulled all the way. Appearance of the next picture was initiated by the participant by moving the joystick back into the central position and pushing the trigger button of the joystick.

To create an unambiguous relation between movements and approach-avoidance behaviors, a zooming effect was used. When the joystick was pushed, the picture became smaller, creating the impression that the picture disappeared in the distance. When the joystick was pulled, the picture became larger, creating the impression that the picture came closer. To this end, seven different sizes of each picture were created: The largest picture filled the screen; then it was reduced six times to 65%, resulting in seven different picture sizes (respectively 100%, 65%, 42%, 27%, 18%, 12% and 7% of the original picture size). Every trial started with the medium-sized picture and the three smaller or larger pictures appeared after pushing or pulling the joystick, respectively.

### Design

Although the groups were comparable with regard to age and educational level (see above), there were large variations within each group in age (from 21 to 55 years) and in educational level (all levels were present in both groups, ranging from finished primary school to a university degree). Therefore, age and educational level were used as covariates in the analyses. The AAT reaction times (RTs) were analyzed according to a 2×2×4 ANCOVA design with the between-subjects factor Group (2: PTSD, Control) and the within-subjects factors Movement (2: push, pull) and Picture Type (4: high-threat sexual, non-threat sexual, high-threat accident, positive). Repeated measures ANCOVAs were used for group comparisons. Where necessary, the Greenhouse-Geisser correction of degrees of freedom was used. Differences between the two groups on PABQ, PSS-SR, BDI, and DES scores were analyzed with ANCOVAs with Group (PTSD, Control) as fixed factor. Pearson correlations were computed to investigate the association of AAT reaction times with PABQ, PSS-SR, BDI, and DES scores.

## Results

### PTSD symptom severity and psychopathology

Means and standard deviations were calculated for each group separately on the PTSD-related psychopathology measures (see Table 1). As expected, PTSD patients scored significantly higher than controls on the PSS-SR, *F*(1,35)=107.24, *p*<.001, partial η^2^=.75; the BDI, *F*(1,35)=53.31, *p*<.001, partial η^2^=.60; and the DES, *F*(1,35)=14.39, *p*=.001, partial η^2^=.29. These results indicate that PTSD patients reported higher levels of PTSD symptom severity, more depression symptoms, and more dissociative experiences than controls.

**Table 1:** Mean questionnaire scores (and standard deviations) per group

	Measure			
	
	PABQ	BDI	DES	PSS-SR
PTSD	55.8 (2.7)	23.7 (9.7)	17.7 (9.9)	29.8 (9.4)
Control	30.3 (2.2)	4.3 (5.0)	7.3 (5.8)	4.4 (4.5)

### Self-reported explicit avoidance

As expected, PTSD patients scored significantly higher than controls on the PABQ, *F*(1,35)=51.9, *p<*.001, partial η^2^=.59. Thus, PTSD patients reported more explicit avoidance behavior.

### Implicit avoidance: AAT

To reduce the influence of outlying data points, trials with a reaction time above 3,500 ms were excluded from the analyses, just like error trials that were finished by moving the joystick completely into the wrong direction. The data of one PTSD patient were completely eliminated because almost half of her responses were errors. For the remaining participants, error rates were low, averaging 6.6%. Mean RTs (and standard errors) per experimental condition are presented in Table 2. The ANCOVA of these RTs revealed the expected significant Group×Movement×Picture type interaction, *F*(2.71, 95)=3.04, *p*=.037, partial η^2^=.08. To explore this interaction in more detail and to test our hypotheses more specifically, we analyzed whether push and pull movements in reaction to the four picture types differed between the two groups. To this end, four Push-minus-Pull RT differences were computed for each participant; called *approach-avoidance scores* (see Table 2). Negative scores indicate faster avoidance than approach and positive values indicate faster approach than avoidance.

**Table 2:** Mean reaction times in ms (with standard errors) and approach-avoidance scores in ms, depending on group, picture type, and movement direction

		Movement		
			
Group	Picture type	Pull	Push	Approach-avoidance score
PTSD	High-threat sexual	888 (34)	823 (28)	−65
	Non-threat sexual	851 (31)	850 (30)	−1
	High-threat accident	837 (28)	801 (26)	−36
	Positive	874 (33)	867 (40)	−7
Control	High-threat sexual	683 (28)	695 (23)	+12
	Non-threat sexual	699 (26)	655 (25)	−44
	High-threat accident	681 (23)	691 (21)	+10
	Positive	713 (27)	699 (33)	−14

These scores were used as the dependent variable in additional ANCOVAs with Group (2: PTSD, control) as between-subjects factor, and age and educational level as covariates. The first ANCOVA revealed that PTSD patients avoided high-threat sexual pictures more than the control participants did, *M*=−65 versus *M*=12, *F*(1,35)=4.56, *p*=.040, partial η^2^=.12. A similar result was also found for high-threat accident pictures, but the difference between PTSD patients (*M*=−36) and control participants (*M*=10) failed to reach statistical significance, *F*(1,35)=3.35, *p*=.076, partial η^2^=.09. These results suggest that PTSD patients, compared with controls, showed implicit avoidance of trauma-related threat pictures, and some of them (see correlations below) also avoided trauma-unrelated threat pictures. In contrast, for both positive pictures and non-threat sexual pictures, the same ANCOVAs did not reveal significant differences between groups, *F*(1,35)=.03, *p*=ns; and *F*(1,35)=1.34, *p*=ns, respectively.

### Correlations of approach-avoidance scores with PABQ, PSS-SR, CAPS, BDI, and DES scores within the PTSD group

As shown in Table 3, all significant correlations were negative, that is, higher questionnaire scores predicted stronger implicit avoidance. Interestingly, however, no significant correlations were found between questionnaires and implicit avoidance of high-threat sexual pictures. This suggests that patients showed implicit avoidance of these most relevant pictures independent of their self-reported explicit avoidance, their symptom severity, their level of depression, and their level of dissociation symptoms (see Table 3). Inspection of the subscales of the PSS-SR and the CAPS revealed only one significant correlation, namely between implicit avoidance of the high-threat sexual pictures and the PSS-SR subscale “arousal,” *r*=−.601, *p*=.014, suggesting stronger avoidance in patients suffering from stronger arousal symptoms.

**Table 3:** Correlations between approach-avoidance scores per picture type and PABQ, PSS-SR, CAPS, BDI, and DES scores in the PTSD group (*N=*16)

	Picture type			
	
Measure	High-threat sexual	Non-threat sexual	High-threat accident	Positive
PABQ	−.34	−.31	−.84**	−.63**
PSS-SR	−.39	−.17	−.50*	−.32
CAPS	−.20	−.14	−.52*	−.45
BDI	−.19	−.73**	−.65**	−.70**
DES	−.27	.01	−.27	.13

Negative values indicate that higher questionnaire scores predict avoidance.

* Correlation is significant at the .05 level.

** Correlation is significant at the .01 level.

In contrast to the rather general avoidance of high-threat sexual pictures, the avoidance of other pictures did depend on several variables. In particular, avoidance of high-threat accident pictures was predicted by high scores on self-reported explicit avoidance (PABQ), both self-reported (PSS-SR) and clinician-rated (CAPS) symptom severity, and by depression (BDI; see Table 3). This also explains why the difference between PTSD patients and controls reported above failed to reach statistical significance: Only patients with higher scores on these questionnaires avoided high-threat accident pictures. In addition, more depressed patients also showed more avoidance of non-threat sexual pictures and of positive pictures. Avoidance of the latter pictures was also predicted by higher levels of self-reported explicit avoidance.

## Discussion

In line with our expectations, the present pilot study indicates that PTSD patients who suffered from sexual trauma, in comparison with controls, show more implicit avoidance of threatening trauma-related pictures. This avoidance was rather general in the patient group tested here. It only varied as a function of self-reported symptoms of arousal: The more arousal PTSD patients experienced, the more they avoided threatening trauma-related pictures. Of special interest is the finding that this implicit avoidance was not associated with self-reported explicit avoidance. This could be viewed as evidence that both avoidance behaviors stem from different underlying processes, and that they may be independent of each other. For instance, PTSD patients may consciously decide to approach a feared stimulus (e.g., during exposure treatment), but still show spontaneous, subtle avoidance movements when encountering the stimulus.

Our explorative question whether the patients would also avoid pictures that are also trauma-related, but non-threatening, and/or pictures that are trauma-unrelated, but threatening, might be answered in favor of the second option. That is, based on our results, one could speculate that at least some PTSD patients, in addition to avoiding threatening trauma-related pictures, also avoid trauma-unrelated, but threatening accident pictures. In contrast to the avoidance of threatening trauma-related stimuli, this avoidance of unrelated threat pictures only occurred in the most burdened patients: It depended on several PTSD-related variables, including level of self-reported explicit avoidance, depression, and PTSD symptom severity. Thus, the more PTSD patients were burdened, the more they also avoided other threatening stimuli, even those unrelated to their trauma. However, it needs to be stressed that the existence of this relation is speculative. Other explanations might account for it; for example, a general anxious disposition among patients with PTSD, resulting in avoidance of all anxiety-provoking materials.

Thus, the present findings suggest that for implicit avoidance to occur, threat level seems to be more important than trauma-relatedness. This is in accordance with generalized attentional bias effects for high-threat words (Litz et al., 1996) and findings of increased amygdala activation, indicating enhanced emotional processing of masked trauma-unrelated fearful faces (Armony et al., 2005; Rauch et al., 2000). However, our results run contrary to previous findings of attentional biases in PTSD, where generalization depended on trauma-relatedness rather than threat level (Cassidy et al., 1992; Fleurkens et al., 2011; Paunovic et al., 2002).

Because we studied a different process, namely implicit avoidance behavior instead of attention, it is difficult to compare the current results to those of the studies mentioned above. Furthermore, we used pictures instead of words, which are processed in different ways, and different trauma groups were used across studies. Interestingly, the present results with a group of sexual violence victims are more in line with earlier studies using non-sexual trauma groups (e.g. Litz et al., 1996). Unfortunately, there are no other published studies about implicit avoidance in PTSD using indirect measures, leaving us with rather minimal possibilities for comparison. It would be worth studying the possible relationship between attentional bias and implicit avoidance. Perhaps attentional bias, accounting for the detection of threat and/or trauma stimuli, precedes processes of implicit avoidance. This would be in line with emotional processing theories (e.g., Foa & Kozak, 1986; Foa et al., 2006), stating that PTSD patients choose to avoid trauma-related materials to protect themselves from anxiety and the feeling of danger. The present study points to the possibility that avoidance may also take place at an *automatic level*, especially in patients with more arousal symptoms. Furthermore, it seems that patients with more self-reported explicit avoidance, symptoms of depression and more severe PTSD symptoms show this implicit avoidance also in response to other threatening stimuli, even if they are trauma-unrelated.

Because our findings are preliminary, and to our knowledge, the first of their kind, further research is needed before firm conclusions can be drawn. Furthermore, the present findings cannot be interpreted without a warning about several limitations of the study. First, it is unclear whether the AAT pictures used here were threatening and trauma-related for all PTSD patients alike, given that they did not rate these themselves. Instead, this rating was done by non-traumatized students in a pilot study. In future studies, individual threat values and trauma-relatedness should be established by the patients themselves. Second, the positive pictures showed images of flowers, children, and happy faces, while the other picture types showed more complex scenes of multiple persons, interactions, and accidents. Although instructions were content-irrelevant, more complex scenes could lead to different implicit reactions as they take longer to process, even in an implicit manner. Third, the present study tested only sexual trauma victims with PTSD, so it is unclear whether the results can be generalized to PTSD patients who experienced other traumas or to non-PTSD patients who experienced sexual violence as well (see also Kimble, Frueh, & Marks, 2009). Future studies should take these limitations into account when studying implicit avoidance behavior in PTSD.

With these considerations in mind, we hope that future studies will shed more light on the possible existence of implicit avoidance in PTSD patients. If future studies repeatedly establish its existence, one could speculate about a few possible clinical applications. In time, if our findings can be replicated, one might try to use implicit avoidance tendencies, and the degree to which they occur for different stimuli, as an additional indicator of PTSD severity before treatment. In line with this, another clinically important question is whether the implicit avoidance of high-threat stimuli remains after treatment, or whether it diminishes with successful treatment, as in spider phobics (see Reinecke, Soltau, Hoyer, Becker, & Rinck, 2012). Moreover, the degree to which implicit avoidance remains present—or remains generalized—might be an independent predictor of relapse after treatment, next to strategic or explicit avoidance. In this sense, implicit avoidance tendencies could be assessed before treatment as a severity measure, and after treatment as an outcome measure that does not rely on subjective self-report.

In addition, more research into the feasibility of *directly* modifying action tendencies by retraining programs is needed. For instance, Wiers et al. (2011) as well as Eberl et al. (2013) demonstrated changed action tendencies in hospitalized alcoholics in accordance with their training condition, with participants in the avoid-alcohol conditions having a lower relapse risk than patients in the control conditions. However, this training involved a re-training of dysfunctional implicit approach tendencies. Whether a similar re-training of implicit avoidance tendencies would be beneficial for PTSD patients would be a highly speculative, but important question for future studies.

Although the role of implicit avoidance behavior in the maintenance of PTSD has been stressed earlier, the present pilot study is actually the first to find both its existence and its occurrence for different types of stimuli. Regarding the latter, although speculative, the present results suggest that level of threat may be more important than mere trauma relatedness. Future studies should shed more light on this issue, and replications of the present preliminary findings are needed.

## Footnotes

^1^Some of the patients (*N=*14) and all controls described in the present study also took part in a study on attentional bias in PTSD, presented elsewhere (Fleurkens et al., 2011).

## Footnotes

^2^The description and results of the EST are reported in Fleurkens et al. (2011).

## Footnotes

^3^The non-threatening vehicle pictures were included because we had anticipated we would also test PTSD patients who had experienced traumatic accidents. However, no such patients could be found and included in the study. Therefore, these pictures are irrelevant to the current research question and excluded from all analyses. They are only mentioned here for reasons of completeness. In contrast, the high-threat accident trauma pictures were included in the study to investigate the possible occurrence of implicit avoidance of another threatening picture type that is unrelated to sexual trauma.
